# Commentary on: Helium Plasma Dermal Resurfacing With and Without Concurrent Aesthetic Surgery of the Face and Neck: A Retrospective Review

**DOI:** 10.1093/asjof/ojac066

**Published:** 2022-08-02

**Authors:** David M Turer

There has been a significant amount of controversy about the use of the Renuvion device (Apyx Medical Corporation, Clearwater, FL) in aesthetic surgery since a warning letter was issued by the FDA in March (Video). However, the Renuvion received 510(k) clearance with a new handpiece for dermal resurfacing in May 2022. This article demonstrates safety and efficacy of the Renuvion for dermal resurfacing both with and without concurrent facial surgery.^1^

The study was a single-site retrospective review of 301 patients, 64% of which included concurrent surgical procedures. Patients had skin types I–IV and on average were treated with 3 passes. The authors collected adverse event (AE) and satisfaction data. Seven percent of patients had minor AEs including prolonged erythema, hyperpigmentation, milia, slow healing, and upper lip hypertropic scarring. There were no serious AEs. Ninety-five percent of patients reported satisfaction with the procedure.

This article is important because it demonstrates the ability to perform aggressive plasma dermal resurfacing with minimal complications. The authors propose that helium plasma resurfacing remains more superficial than other modalities, which may account for the relatively low number of AEs. Recovery after the procedure is similar to other deep resurfacing techniques, such as full-field Erbium laser resurfacing.

The patients in their series had relatively short follow-up, on average, roughly two months for the satisfaction data. I would like to see the patients followed out longer. Additionally, patient satisfaction was judged by comments on the chart, not using a standard assessment. A future prospective study should include formal evaluation of both efficacy and patient satisfaction.

Many plastic surgeons already use the Renuvion for subdermal skin tightening, and by using the new handpiece; this gives them the option to perform dermal resurfacing as well without any other new equipment. I would like to commend the authors for sharing their experience and would be interested to learn more from them about the subtleties of performing the treatment, for example, how they determine the appropriate number of passes and their current preoperative and postoperative regimens.
